# Caregiver Strain and Perceived Impact of COVID‐19 on Dementia Caregiving

**DOI:** 10.1002/alz70861_108706

**Published:** 2025-12-23

**Authors:** David R Lee, Xi Zhu, Ron D Hays, Jenny Summapund, Thomas M. Gill, Peter Peduzzi, Gery W. Ryan, Ryan Baxter‐King, Katy LB Araujo, Erich J Greene, Lee Jennings, Jeff Williamson, Alan B Stevens, Maya L Lichtenstein, Rebecca Galloway, David B. Reuben

**Affiliations:** ^1^ UCLA Multicampus Program in Geriatric Medicine and Gerontology, Los Angeles, CA USA; ^2^ UCLA Fielding School of Public Health, Los Angeles, CA USA; ^3^ UCLA Division of General Internal Medicine and Health Services Research, Los Angeles, CA USA; ^4^ David Geffen School of Medicine at UCLA, Los Angeles, CA USA; ^5^ Yale University, New Haven, CT USA; ^6^ Yale School of Public Health, New Haven, CT USA; ^7^ Kaiser Permanente, School of Medicine, Pasadena, CA USA; ^8^ UCLA Department of Political Science, Los Angeles, CA USA; ^9^ Oklahoma University, Okalhoma City, OK USA; ^10^ Wake Forest University School of Medicine, Winston‐Salem, NC USA; ^11^ Baylor Scott & White Health, Temple, TX USA; ^12^ Geisinger Health System, Wilkes Barre, PA USA; ^13^ University of Texas Medical Branch, Galveston, TX USA

## Abstract

**Background:**

The COVID‐19 pandemic disproportionately affected people with Alzheimer’s disease and related dementias and their caregivers. Caregivers with high strain may have been particularly vulnerable to disruptions caused by COVID‐19. We examined the relationship between caregiver strain and perceived impact of COVID‐19 on dementia caregiving, and whether this relationship is moderated by county‐level COVID‐19 burden.

**Method:**

We included caregivers from a RCT of dementia care models (The D‐CARE Study) who completed an 8‐item COVID‐19 questionnaire at their 3‐month follow‐up visit. The exposure was baseline caregiver strain, measured using the Modified Caregiver Strain Index, with scores ≥13 indicating high strain. The outcome was perceived impact of COVID‐19 on dementia caregiving, measured using a composite score from the COVID‐19 questionnaire ranging from 0 (no impact) to 10 (maximum impact). The potential moderator was county‐level COVID‐19 burden, measured as average daily deaths per 100,000 people over the 30 days preceding COVID‐19 questionnaire completion. We used an adjusted Two‐Part Model, including: 1) logistic regression to assess whether the caregiver perceived an impact of COVID‐19 on caregiving (yes/no) and 2) linear regression to examine the severity of the perceived impact among participants who reported experiencing an impact (non‐zero responses).

**Result:**

Of 1,073 caregivers, 33% reported high baseline strain. High‐strain caregivers were more often younger, female, unmarried, non‐White, and family members; they were also more likely to report worse health and to have been a caregiver for >5 years as compared to low‐strain caregivers (all *p* <0.01). High‐strain caregivers had a 60% increase in the odds of reporting a perceived impact of COVID‐19 on dementia caregiving (OR = 1.6, 95% CI: 1.1–2.3, *p* =0.013). Among caregivers who reported being impacted by COVID‐19 (non‐zero response), on average, caregivers with high strain had a 1.2‐point higher perceived impact of COVID‐19 on dementia caregiving (95% CI: 0.9–1.5, *p* <0.001). The county‐level COVID‐19 burden did not significantly moderate these relationships.

**Conclusion:**

Caregivers with pre‐existing high strain may be especially vulnerable to the adverse impacts of unexpected public health emergencies such as COVID‐19 and could benefit most from targeted interventions to foster resilience.

